# Moxibustion Improves Chronic Heart Failure by Inhibiting Autophagy and Inflammation via Upregulation of mTOR Expression

**DOI:** 10.1155/2021/6635876

**Published:** 2021-02-02

**Authors:** Qingling Li, Wei Wang, Qiang Ma, Ran Xia, Bing Gao, Guoqi Zhu, Jing Wang

**Affiliations:** ^1^Graduate School, Anhui University of Chinese Medicine, Hefei 230012, China; ^2^School of Chinese Medicine, Anhui University of Chinese Medicine, Hefei 230012, China; ^3^Key Laboratory of Xin'an Medicine of Ministry of Education, Anhui University of Chinese Medicine, Hefei 230038, China

## Abstract

How moxibustion improves chronic heart failure is extremely complex and still unclear. This study aimed to explore whether moxibustion inhibits autophagy and reduces inflammation by regulating mTOR expression to induce myocardial protective effects and alleviate symptoms associated with chronic heart failure. Echocardiography was used to detect cardiac function and cardiac structure of rats, including heart rate (HR), left atrium diameter (LA), left ventricular diameter (LV), left ventricular posterior wall (LVPW), interventricular septum (IVS), ejection fraction (EF), and fractional shortening (FS). BNP and NT-pro BNP levels were measured by enzyme-linked immunosorbent assay (ELISA). Autophagy-associated protein (ATG) genes and mTOR were detected by PCR. The expression of mTOR and phosphorylated-mTOR was detected through western blotting of proteins from myocardial tissue samples. The left ventricular inflammatory response was detected by immunohistochemistry and included ICAM-1, VCAM-1, MMP-2, and MMP-9 expression. The relationship between autophagy and inflammation was analyzed by correlation analysis. The results from echocardiography and ELISA showed that moxibustion could significantly improve heart function and structure. Western blot and PCR results showed that moxibustion treatment elevated mTOR expression. Further, moxibustion could inhibit autophagy and regulate the expression of key autophagy-related genes, including Vps34, ATG3, ATG5, ATG7, ATG12, and ATG13. By contrast, rapamycin could partially reduce the effects of moxibustion. Immunohistochemistry results indicated that moxibustion could reduce myocardial inflammation. Moreover, there was a positive correlation between autophagy and inflammation. Moxibustion can protect cardiac function in rats with heart failure, possibly inhibiting excessive autophagy of cardiomyocytes and reducing inflammatory reactions through the elevation of mTOR expression.

## 1. Introduction

Chronic heart failure (CHF) is the ultimate outcome of different cardiovascular diseases, which represents a public health problem requiring urgent attention all over the world due to its high morbidity, mortality, and rehospitalization rates [1, 2]. The 2018 China Cardiovascular Diseases Report indicated that the number of patients with cardiovascular disease exceeded 200 million, and patients with heart failure accounted for 2%. Furthermore, the prevalence and mortality of CHF are still on the rise and CHF has become a disease that seriously threatens human life and health [3]. In view of the fact that several current treatment methods exert adverse side effects on patients and patients exhibit poor response to treatments, finding a safe complementary and alternative therapy is essential for the management of CHF.

Autophagy is a protective mechanism through which cells strive to survive under adverse conditions. Conversely, excessive autophagy may lead to accelerated cell death, resulting in a variety of acute and chronic diseases [4, 5]. Based on accumulating evidence on CHF, it has been proposed that disordered and reduced autophagy is involved with its progression [6, 7]. mTOR, an atypical serine/threonine PI3K-related kinase family, is involved in diverse cellular processes, such as cellular growth, proliferation, survival, protein synthesis, autophagy, and metabolism [8]. In addition, the nutrient sensor mTOR is likely the core autophagy regulator [9–11] and is the central regulator of physiological and pathological processes in the cardiovascular system. Autophagy is regulated by autophagy-related genes (ATG), which promotes the formation of phagosomes [12]. Thus, targeting autophagy has become a new strategy for the treatment of CHF.

CHF is associated with inflammation [13], which is characterized by chronic low-grade vascular inflammation [14]. The effect of ICAM-1 and VCAM-1 has been associated with the prognosis of patients with CHF. In particular, VCAM-1 can be used for risk screening of patients with CHF [15]. Matrix metalloproteinases (MMPs) are key pathophysiological markers of CHF [16] whose activity is related to cardiac remodeling and left ventricular configuration of heart disease [17]. Some studies have shown that ventricular remodeling is accompanied by an increase in MMP-2 and MMP-9 activity [18, 19].

Moxibustion, a treatment derived from traditional Chinese medicine (TCM), has been widely applied for treating various diseases, including cardiovascular disease, and exhibits good therapeutic effects [20, 21]. Currently, studies evaluating the activities of moxibustion have mainly focused on its anti-inflammatory effects, but less attention has been paid to its inhibitory effects on autophagy in CHF. Thus, in this study, we evaluated whether moxibustion could improve CHF and whether moxibustion mediates autophagy-inflammation to improve cardiac function and structure by elevating mTOR expression using an *in vivo* animal model. Our objective was to explore the molecular mechanisms of moxibustion in the treatment of CHF.

## 2. Materials and Methods

### 2.1. Animals and Treatment Groups

This study was performed in accordance with the animal welfare regulations for the Anhui Medical University (Anhui, China). A total of 90 male Sprague-Dawley (SD) rats weighing 200–300 g were obtained for this study, and all were maintained under specific-pathogen-free (SPF) conditions (Animal license-number SYXK [Anhui] 2017–006). All experimental procedures were approved by the Animal Ethics Committee of the Anhui University of Chinese Medicine. The rats were kept in separate cages (Kangwei IR60), with free access to air, food, and water. During the experiment, the handling of animals was in accordance with the guidelines for the handling of Experimental Animals issued by the Ministry of Science and Technology. Every effort was made to minimize the pain and discomfort to the animals; animals were euthanized using 3% pentobarbital sodium at a dose of 30 mg/kg intraperitoneal injection at the end of the experimental procedures.

To explore the effects of moxibustion on cardiac structure and function in rats with CHF, rats were randomly divided into three groups: the control group, model group (Adriamycin- (ADR-) induced) and moxibustion group (ADR plus moxibustion treatment). We observed changes in the heart rate (HR), left atrium diameter (LA), left ventricular diameter (LV), left ventricular posterior wall (LVPW), interventricular septum (IVS), ejection fraction (EF), and fractional shortening (FS) via echocardiographic evaluation of cardiac structure and function. Serum BNP and NT-pro BNP levels were assayed using ELISA kits (Elabscience Biotechnology Co., Ltd., China). To assess whether moxibustion mediates the autophagy-inflammation effects on CHF through elevating mTOR expression, rats were randomly divided into five groups: the control group, model group (ADR-induced CHF), rapamycin [RAPA] + moxibustion group (ADR plus RAPA-moxibustion treatment), RAPA group (ADR plus RAPA-treatment), and moxibustion group (ADR plus moxibustion-treatment). We observed changes in mTOR expression in myocardial tissue, in expression of autophagy-related genes Vp34, ATG3, ATG5, ATG7, ATG12, and ATG13, and in inflammatory factors ICAM-1, VCAM-1, MMP-2, and MMP-9. We also examined the correlation between autophagy and inflammation.

### 2.2. Animal Model and Treatment

**Figure 1:**
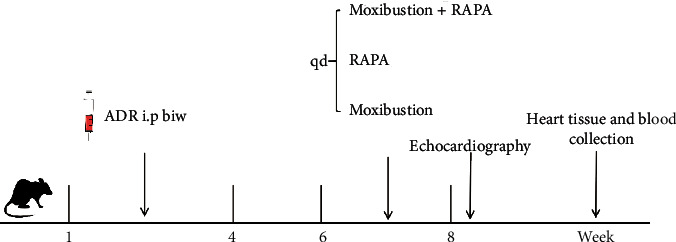
The flow diagram of experimental protocol. i.p.: intraperitoneal injection; biw: biweekly; qd: quaque die; ADR : adriamycin; RAPA: rapamycin.

The rat model of CHF is based on the assumption of induction of myocardial toxicity by ADR [22]. ADR was injected intraperitoneally to produce a CHF rat model: ADR and 0.9% saline solution were used to prepare the injection solution to a concentration of 1 mg/mL. A single injection of 2 mg/kg was given twice weekly for 4 weeks, with a cumulative injection volume set at 16 mg/kg. The intervention treatment was started during the sixth week. Rats in the control and model groups were placed on the rat platform once a day without any intervention. Rats in the RAPA group were intraperitoneally injected with RAPA (Sigma, USA) 2 mg/kg once a day for 3 weeks and placed on the rat platform once a day. RAPA is a potent and specific mTOR inhibitor and an autophagy activator [23]. In the moxibustion group, rats were placed on the rat platform and performed on Feishu (BL13, 7 mm below the third thoracic spinous process on both sides) and Xinshu (BL15, 7 mm below the fifth thoracic spinous process on both sides) points [24] with moxibustion sticks (5 mm × 120 mm, produced by Wolong Traditional Chinese Medicine Moxibustion Factory, Nanyang). The suspended moxibustion was performed for 20 min at the distance of 2 cm directly above the acupoints once a day for 3 weeks, with the skin temperature of acupoints reaching 44 ± 1°C [25]. The RAPA + moxibustion group received moxibustion as the base treatment and then were given RAPA 2 mg/kg intraperitoneal injections once a day for 3 weeks. The mortality rate in rats was 30% across the 8-week experiment period, with most of the deaths occurring during the first four weeks of modeling, which were likely related to acute pump failure or to fatal arrhythmias [26]. A schematic diagram of the experimental protocol is shown in Figure 1.

### 2.3. Echocardiographic Assessment

Intraperitoneal injection of 3% pentobarbital sodium was used for anesthesia at a dose of 30 mg/kg, and the left ventricular cardiac functions were noted via echocardiography in the 2D B-mode and M-mode (Siemens Acuson Oxana 3; USA). HR, LA, LV, LVPW, IVS, EF, and FS were tested for at least three nonstop cardiac cycles. At the end of the measurements, all experimental rats were anaesthetized with sodium pentobarbital and euthanized to obtain blood and heart tissue samples.

### 2.4. Enzyme-Linked Immunosorbent Assay

Levels of serum BNP and NT-pro BNP were measured by enzyme-linked immunosorbent assay kits (ELISA, Elabscience Biotechnology Co., Ltd., China) according to the manufacturer's protocols. First, a 50 *μ*L volume of supernatant was added to each well and was incubated for 2 h at room temperature and then biotin antibody was added to each well and incubated for 1 h. Next, horseradish peroxidase was added to the wells and incubated for an additional 30 min. Subsequently, the substrate reagent was added to each well. The absorbance at 450 nm was immediately detected by a microplate reader (iMark Microplate Reader, BIO-RAD, China).

### 2.5. Western Blotting Analysis

Myocardial tissues were extracted from the left ventricle, and protein was extracted from the homogenized tissue using precooled RIPA lysis solution (Shanghai Beyotime Institute of Biotechnology, China) supplemented with 1% phenylmethylsulphonyl fluoride (Shanghai Beyotime Institute of Biotechnology, China). The BCA (Beijing Solarbio Science and Technology Co., Ltd., China) method was used to determine protein concentrations. To investigate the expression of mTOR and its phosphorylated protein (p-mTOR) in heart tissues, 50 mg of protein was separated by SDS-PAGE and transferred to PVDF membranes, which were blocked with 5% skim milk. The membranes were incubated with primary antibodies at 4°C overnight. The primary antibodies used were anti-mTOR (1 : 2000, ab137133; Abcam, United States), anti-phospho-mTOR (1 : 5000, ab109268; Abcam, United States), anti-GAPDH (K106389P; Beijing Solarbio Science and Technology Co., Ltd., China), and IgG-HRP (SE134; Beijing Solarbio Science and Technology Co., Ltd., China). The membranes were then incubated with the appropriate secondary antibody for 1 h at room temperature and exposed to ECL (Beijing Solarbio Science and Technology Co., Ltd., China) in the darkroom at room temperature.

### 2.6. Real-Time Quantitative Polymerase Chain Reaction

We used the reverse transcriptase polymerase chain reaction (RT-PCR) to detect mRNA expression of mTOR, Vp34, autophagy-related gene (ATG) 3 (ATG3), ATG5, ATG7, ATG12, and ATG13 in left ventricular tissues. The total RNA from each sample was extracted using Trizol Reagent (B5 11321-UNIQ-10, Sangon Biotech, China), and then the RNA was retrotranscribed to cDNA using the EasyScript One-Step gDNA Removal and cDNA Synthesis SuperMix (TransGen Biotech, China). The reaction volume was 20 *μ*L (2 *μ*L cDNA, 0.5 *μ*L forward and reverse primers each, 10 *μ*L Mix, and 7 *μ*L dH_2_O). To analyze the cDNA of Vp34, ATG3, ATG5, ATG7, ATG12, and ATG13, PCR conditions were 15 s at 95°C for denaturation and 1 min at 55°C for annealing and extension. Each gene was amplified by 40 cycles. To determine the relative quantities of mTOR, Vp34, ATG3, ATG5, ATG7, ATG12, and ATG13 mRNA, GAPDH was used as the internal standard. The primer sequences are reported in Table 1.

### 2.7. Immunohistochemistry

Samples from the left ventriculum were fixed in 4% paraformaldehyde at 25°C, embedded in paraffin, and cut into 3 *μ*m thick sections. After deparaffinization in xylene, the tissues were subjected to antigen retrieval using 10 mM citrate-phosphate buffer (pH 6.0) and incubated in 3% H_2_O_2_ for 25 min. Then, the sections were blocked with 1% bovine serum albumin in PBS for 10 min. Following incubation with a prediluted biotinylated pan-specific universal secondary antibody (Servicebio, China) for 50 min at room temperature, sections were incubated overnight with mouse monoclonal ICAM-1 (1 : 800, Servicebio), VCAM-1 (1 : 400, Servicebio), MMP-2 (1 : 2000, Servicebio), and MMP-9 (1 : 800, Servicebio) antibody at 4°C. The tissues were washed with PBS, incubated in the streptavidin/peroxidase complex for 5 min and then washed with PBS again, before being washed with 3, 3′-diaminobenzidine (Servicebio, China) for 7 min. Sections were stained with hematoxylin for 3 min, then washed, and mounted. Images were captured using a fluorescent microscope (XSP-C204, CIC, Germany).

### 2.8. Correlation Analysis

Pearson correlation coefficients were used to compare levels of ICAM-1, VCAM-1, MMP-2, MMP-9 with Vp34, ATG3, ATG5, ATG7, ATG12, and ATG13 in left ventricular tissues, respectively. Plots were generated using the statistical programming language *R* version 4 software (Inc., USA).

### 2.9. Statistical Analysis

Data are expressed as mean ± standard deviation (SD). For parametric data, the comparison of different groups was performed by one-way analysis of variance (ANOVA), followed by Tukey's post hoc test for multiple comparisons. Statistical analyses were performed using GraphPad Prism version 6 software (Inc., San Diego, CA). All results were considered statistically significant at *p* < 0.05, *p* < 0.01, and *p* < 0.001.

## 3. Results

### 3.1. Moxibustion Affected CHF Cardiac Structure and Function

**Figure 2:**
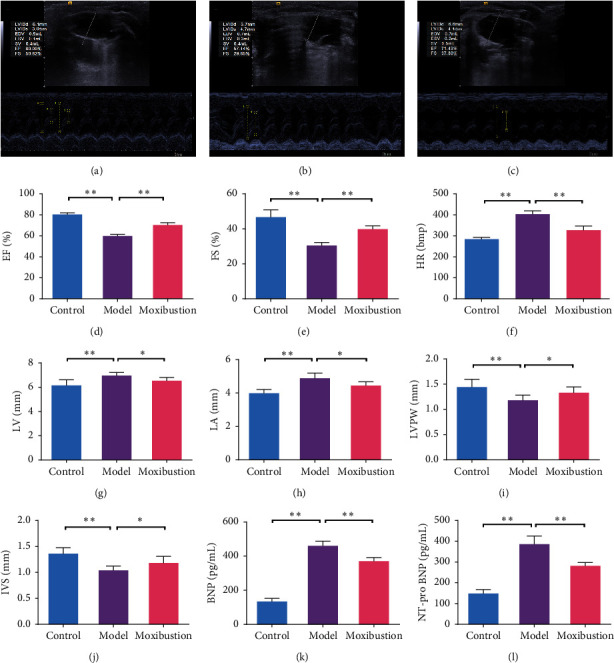
Moxibustion improves chronic heart failure cardiac structure and function. Representative M-mode echocardiograms were recorded. (a) Control. (b) Model. (c) Moxibustion. Echocardiography was carried out. (d) EF. (e) FS. (f) HR. (g) LV. (h) LA. (i) LVPW. (j) IVS. BNP (k) and NT-pro BNP (l) levels of the rat serum were determined using ELISA kit. All data are presented as mean ± SD from three independent experiments (*n* = 7–9). ^*∗*^*p* < 0.05; ^*∗∗*^*p* < 0.01.

After the 8-week experimental period, echocardiography revealed that the CHF rat model had been successfully established, based on evidence of the marked reduction in EF and FS values in the model group compared to the control group (*p* < 0.01, Figures 2(d) and 2(e)). Further, levels of HR, LV, LA, BNP, and NT-pro BNP were significantly increased in the model group compared to the control group (*p* < 0.01, Figures 2(f)–2(h), 2(k), and 2(l)). At the same time, LVPW and IVS significantly decreased in the model group when compared with those in the control group (*p* < 0.01, Figures 2(i)–2(g)), which indicated that cardiac dysfunction and structural alterations had occurred. Compared to the model group, the values of EF and FS were significantly increased in the moxibustion-treated group (*p* < 0.01, Figures 2(d) and 2(e)). HR and serum BNP and NT-pro BNP concentrations in the moxibustion group were significantly decreased (*p* < 0.01, Figures 2(f), 2(k), and 2(l)). Likewise, LV and LA in the moxibustion-treated group were also decreased (*p* < 0.05, Figure 2(g)–2(h)) when compared with those of the model group. In contrast, LVPW and IVS in the moxibustion-treated group were increased (*p* < 0.05, Figures 2(i)–2(g)) when compared with those of the model group.

### 3.2. Moxibustion Increased mTOR Expression in the CHF Rat Model

**Figure 3:**
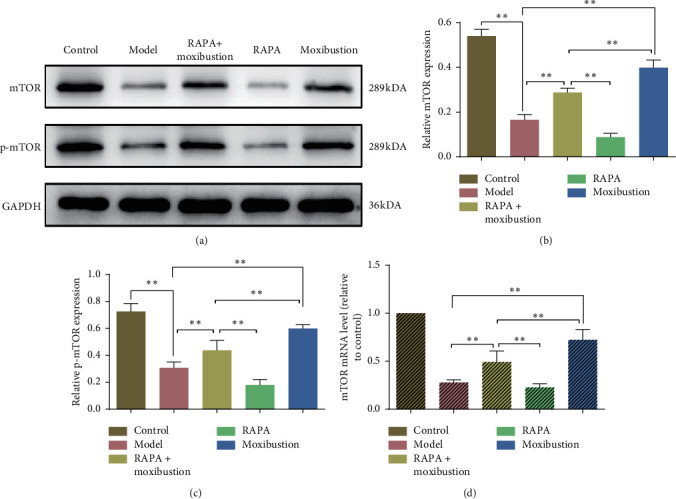
Moxibustion increases mTOR expression in chronic heart failure rats. (a) Immunoblot levels of mTOR, p-mTOR in rats of left ventricular tissues in each group. mTOR (b) and p-mTOR (c). Protein levels were measured using western blot analysis. mTOR mRNA level was measured using qRT-PCR analysis (d). All data are presented as mean ± SD from three independent experiments (*n* = 7–9). ^*∗∗*^*p* < 0.01.

Western blotting demonstrated that the relative expression of mTOR and p-mTOR in the ADR-model group was significantly downregulated compared to the control group (*p* < 0.01, Figures 3(b) and 3(c)). Compared to the model group, the relative expression of mTOR and p-mTOR was significantly upregulated in the moxibustion group and RAPA + moxibustion group (*p* < 0.01, Figures 3(b) and 3(c)). Compared with the RAPA groups, the relative expression of mTOR and p-mTOR was significantly increased following RAPA + moxibustion treatment (*p* < 0.01, Figures 3(b) and 3(c)). Compared with the moxibustion groups, the relative expression of mTOR and p-mTOR was significantly decreased following RAPA + moxibustion treatment (*p* < 0.01, Figures 3(b) and 3(c)). Further, qRT-PCR showed that mTOR expression was significantly upregulated in the moxibustion and RAPA + moxibustion groups (*p* < 0.01, Figure 3(d)) compared to the model group. Moreover, compared to the RAPA group, the expression of mTOR was significantly upregulated in the RAPA + moxibustion group (*p* < 0.01, Figure 3(d)), while when compared to the moxibustion group, the mTOR expression was significantly downregulated in the RAPA + moxibustion treatment group (*p* < 0.01, Figure 3(d)).

### 3.3. Moxibustion Affected Autophagy in the CHF Rat Model

**Figure 4:**
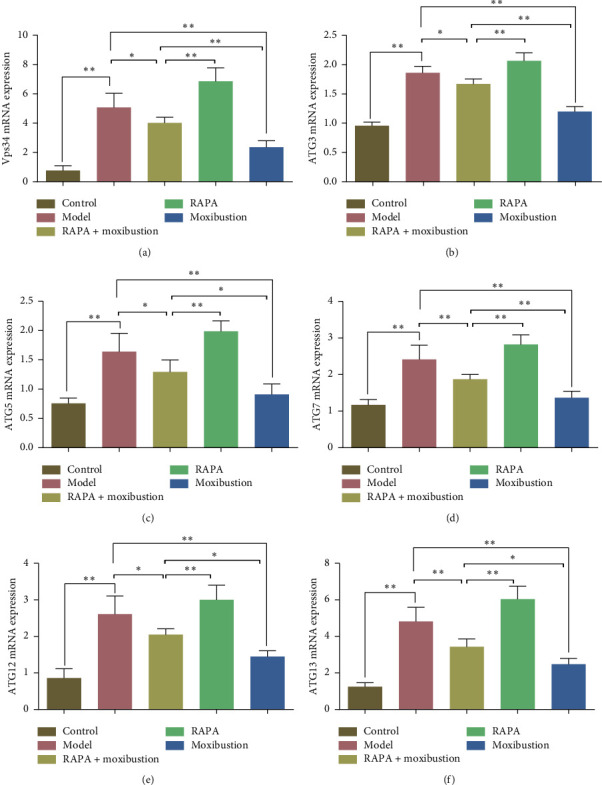
Moxibustion effects the gene expression of autophagy-associated genes in chronic heart failure rats. Autophagy-associated genes, including Vps34 (a), ATG3 (b), ATG5 (c), ATG7 (d), ATG12 (e), and ATG13 (f) were examined via RT-PCR analysis. All data is presented as mean ± SD from three independent experiments (*n* = 7–9). ^*∗*^*p* < 0.05; ^*∗∗*^*p* < 0.01.

The moxibustion effects on autophagy-related genes were studied by PCR. The results demonstrated that the transcriptional expression of Vps34, ATG3, ATG5, ATG7, ATG12, and ATG13 in the model group was significantly upregulated compared to those of the control group (*p* < 0.01, Figures 4(a)–4(f)). However, when compared to the model group, the expression of these autophagy-related molecules was significantly downregulated following treatment with moxibustion (*p* < 0.01, Figures 4(a)–4(f)), which indicated that moxibustion could inhibit autophagy. Compared with the model group, ATG7 and ATG13 expression were significantly downregulated in the RAPA + moxibustion group (*p* < 0.01, Figures 4(d) and 4(f)), as was the expression of Vps34, ATG3, ATG5, and ATG12, albeit with lower statistical significance (*p* < 0.05, Figures 4(a)–4(c), 4(e)). Compared to the RAPA group, the expression for these autophagy-related genes was significantly downregulated in the RAPA + moxibustion group (*p* < 0.01, Figures 4(a)–4(f)). Levels of Vps34, ATG3, and ATG7 expression observed in the moxibustion group were significantly upregulated by RAPA + moxibustion treatment (*p* < 0.01, Figures 4(a), 4(b), and 4(d)), as was the expression of ATG5, ATG12, and ATG13 (*p* < 0.05, Figure 4(c), 4(e), and 4(f)).

### 3.4. Moxibustion Affected Inflammatory Factors in the CHF Rat Model

**Figure 5:**
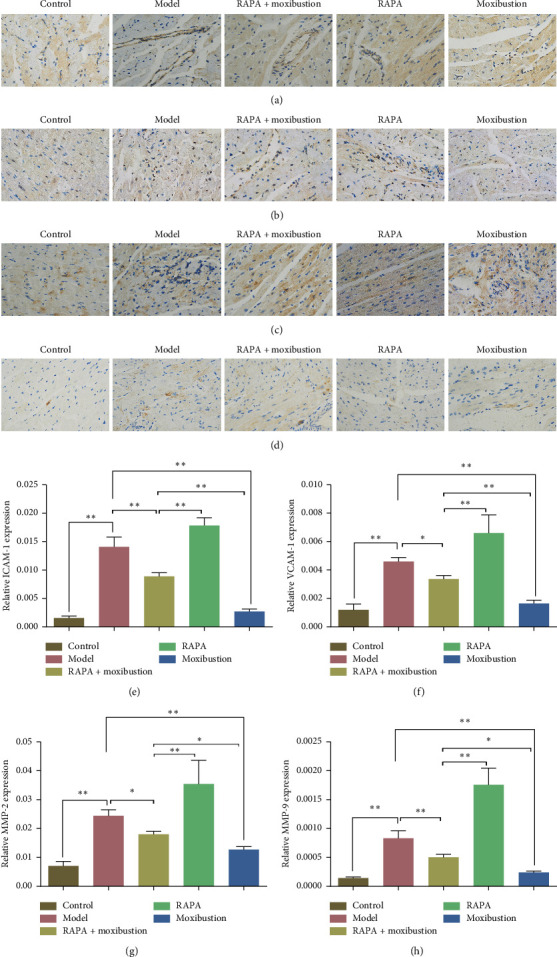
Moxibustion inhibited inflammatory factors in the CHF rat model (original magnification: ×400). Representative images of ICAM-1 (a), VCAM-1 (b), MMP-2 (c), and MMP-9 (d). Quantification data of ICAM-1 expression (e), VCAM-1 expression (f), MMP-2 expression (g), and MMP-9 expression (h). All data are presented as means ± SD from three independent experiments (*n* = 7–9). ^*∗*^*p* < 0.05; ^*∗∗*^*p* < 0.01.

Immunohistochemistry was used to assess anti-inflammatory effects induced by moxibustion. In the ADR model group, the expression of ICAM-1, VCAM-1, MMP-2, and MMP-9 was significantly increased compared to the control group (*p* < 0.01, Figures 5(e)–5(h)), while their expression was all significantly decreased by moxibustion treatment when compared to the model group (*p* < 0.01, Figures 5(e)–5(h)), which demonstrated that moxibustion induced anti-inflammatory effects. In addition, compared with the ADR model group, the expression of ICAM-1 and MMP-9 was significantly lower in the RAPA + moxibustion treatment group (*p* < 0.01, Figure 5(e), 5(h)), as was the expression of VCAM-1 and MMP-2 (*p* < 0.05, Figures 5(f) and 5(g)), whereas, compared to the RAPA group, the expression of inflammatory factors was significantly higher in the RAPA + moxibustion group (*p* < 0.01, Figures 5(e)–5(h)). Compared to the moxibustion group, the expression of ICAM-1 and VCAM-1 was significantly increased by RAPA + moxibustion treatment (*p* < 0.01, Figures 5(e) and 5(f)), as was the expression of MMP-2 and MMP-9 (*p* < 0.05, Figures 5(g) and 5(h)).

### 3.5. Correlation Analysis

**Figure 6:**
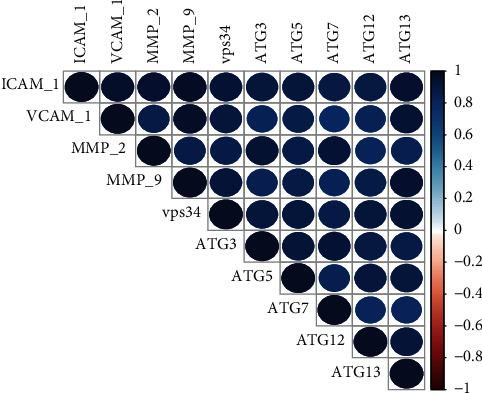
Correlation analysis. R language was used to draw a correlogram chart. Positive correlations are displayed in blue and negative correlations in red. Color intensity and the size of the circle are proportional to the correlation coefficients (*n* = 390).

Correlation analysis was performed between autophagy-associated genes (Vps34, and ATG3, *5*, *7*, 12, and 13) and inflammatory factors (ICAM-1, VCAM-1, MMP-2, MMP-9), respectively (Figure 6). The expression of each autophagy-associated genes showed a strongly positively and significant correlation with the expression of each inflammatory factor (for all correlations, *p* < 0.001).

## 4. Discussion

Moxibustion is an important component of traditional Chinese medicine and is utilized to prevent and treat a variety of chronic diseases. It has been increasingly applied in health preservation and the effectiveness and safety of moxibustion for the treatment of different diseases have been evaluated. For example, a pilot controlled clinical trial concluded that moxibustion was effective in modulating heart rate variability over the long term [27]. Moxibustion and acupuncture have been utilized for treating heart failure primarily in combination with pharmacological agents from western medicine [28]. Studies have shown that moxibustion can improve major heart function indicators, such as HR, LVEF, EF, BNP, and NT-pro BNP in patients with HF [29].

In our previous studies examining moxibustion for the treatment of CHF, we also found that moxibustion played a key role in myocardial protection by regulating the neuroendocrine-immune response, by inhibiting excessive autophagy, and by improving myocardial hypertrophy and cardiac functions [30–32]. Considering this evidence, herein, we examined the intracellular mechanisms involved in moxibustion regulation of cardiomyocyte autophagy and in improvement of ventricular remodeling. Our results demonstrated that RAPA could still completely block the effects of moxibustion on autophagy and inflammation in CHF rats, which might explain that moxibustion might also activate other cell signaling pathways to promote cardiac function. For example, Zhang et al. [33] found that moxibustion was likely to regulate the expression of adenosine 5‘-monophosphate- (AMP-) activated protein kinase to recover myocardial tissue in rats with exhaustive exercise. Further, Tan et al. [34] confirmed that moxibustion had a protective effect in rats with ischemic myocardium, which is probably related to downregulating expression of LC3 I/II and Beclin 1. Traditional Chinese medicine has made great progress in the treatment of CHF, due to the advantages of multipathway, multitarget, and mild adverse reactions.

As a distinct phenomenon of eukaryotic cells, autophagy is characterized by degradation and recycling of intracellular biological macromolecules and damaged organelles [35, 36]. Autophagy degradation products provide energy requirements to maintain cellular function, which assists in cell survival. However, the overexpression of autophagy may also lead to cell damage and death [37]. The mTOR signaling pathway is an essential regulator of cardiac autophagy and plays a critical role in regulating the development and physiological growth of the heart [38]. In addition, mTOR is an essential negative regulator of autophagy, involved in the regulation of the initiation and termination of autophagy processes by controlling Vps34 and other intracellular activities [39]. The signaling complex 1 (mTORC1) is a multiprotein complex including mTOR that participates in the autophagy pathway and leads to the inhibition of autophagy through the phosphorylation of multiple autophagy-related proteins, such as ATG13 [40].

Several key autophagy genes, including Vps34, ATG3, ATG5, ATG7, ATG12, and ATG13, are involved in the autophagy process [41]. Vps34 activity is regulated by the cardiac autophagy regulation center and is present in controlling autophagy at multiple stages [42]. Three important protein complexes are presented in autophagy induction, which can dephosphorylate ATG13 and bind ATG1 to promote autophagy. In addition, ATG3, ATG5, ATG7, and ATG12 interact with autophagy effectors to initiate biogenesis in autophagosomes [43–45]. Evidence has also been provided indicating that the inhibition of autophagy is beneficial to the heart during the decompensation phase of heart failure [46, 47]. The regulation of key autophagy proteins, such as ATG5 and ATG7 in the heart, suggests that autophagy can prevent pathological remodeling and dysfunction [48]. In our study, we demonstrated that autophagy levels increased in the CHF model, which was manifested by the increase in expression of Vps34, ATG3, ATG5, ATG7, ATG12, and ATG13. By regulating the expression of these key proteins in the autophagy process, moxibustion can reduce autophagy in the HF model. In our CHF model, moxibustion protected against heart injury by inhibiting autophagy.

The mechanisms involved in CHF are complex, and many are still unclear, although various theories have been proposed. According to the cytokine hypothesis, the progression of CHF is mediated by a variety of cytokines, which induce cardiomyocyte hypertrophy and eventually lead to poor ventricular remodeling [49]. Some studies have reported that inflammatory cytokines play an important role in the development of CHF [50]. Levels of all inflammatory cytokines increase when the myocardium is damaged and continue to rise during CHF [51, 52]. Recent studies have provided evidence that inflammatory cytokines and markers of inflammation such as soluble adhesion molecules are elevated in the plasma of patients with CHF [53]. ICAM-1 binding to integrins produces proinflammatory effects, and VCAM-1 has been reported to be involved in developing atherosclerosis [54]. Similarly, among the MMPs family, the most frequently analyzed are gelatinases, such as MMP-2 and MMP-9, which produce matrix factors subsequent to proinflammatory stimulation and are associated with heart failure syndrome [55, 56].

Autophagy and inflammation are functionally interrelated and lead to the induction of autophagy-inflammatory reactions [57]. Under normal circumstances, autophagy can inhibit inflammation and produces an anti-inflammatory response by removing damaged or senescent organelles, and, in turn, inflammation can activate autophagy [58, 59]. Many studies have shown that autophagy can regulate the expression of inflammatory factors, influence the development of inflammatory cells, and is itself affected by cytokine activity [60]. For example, Racaneli et al. [61] showed that autophagy could be critical for inhibiting inflammation at baseline; however, persistent or inefficient autophagy could promote injury when not regulated. Furthermore, Wang et al. [62] demonstrated that activation mTOR and inhibition of autophagy suppressed cigarette smoke-induced airway inflammation. However, Zhou et al. [63] showed that boosting the activity of mTOR-dependent autophagy stimulators might improve experimental colitis. Overall, these studies suggest that mTOR-independent autophagy should also be considered.

## 5. Conclusions

In conclusion, the present study provides evidence supporting moxibustion treatment for improving cardiac structure and function of CHF. The underlying mechanism may involve the regulation of autophagy and enhanced anti-inflammatory responses by inducing mTOR expression.

## Figures and Tables

**Table 1:** Nucleotide sequences of primers used in real-time PCR.

Gene	Primers (5'-> 3′ sequences)
Vp34	GGAACTTATCCCGTTGCCTT
	ATTTGCCTCCATCTTCCGTC
ATG3	GACGCCATTCTGCAAACAAGA
	GTTAAAGGCTGCCGTTGCTC
ATG5	ACGGATTCCAACGTGCTTTAC
	AGGGGTGTGCCTTCATATTCA
ATG7	GACCTTCGCGGACCTAAAGA
	TGACGCCTTCAGTTCGACAC
ATG12	CTGCTGAAGGCTGTAGGAGA
	AGGGGCAAAGGACTGATTCAC
ATG13	TGTGGGGCGATCTATGTGTG
	CAGCAGCAGTGACAATCGGT
mTOR	CAGACGCCAATGAGAGGAAG
	CACTTGAGGGGAGGAGGTTC
GAPDH	TCTATCCTGGCCTCACTGTC
	CAGTCCGCCTAGAAGCATTTG

## Data Availability

The datasets used and/or analyzed during the current study are available from the corresponding author on reasonable request.
